# Stripe-Patterned Al/PDMS Triboelectric Nanogenerator for a High-Sensitive Pressure Sensor and a Novel Two-Digit Switch with Surface-Edge Enhanced Charge Transfer Behavior

**DOI:** 10.3390/nano15100760

**Published:** 2025-05-19

**Authors:** Chung-Yu Yu, Chia-Chun Hsu, Chin-An Ku, Chen-Kuei Chung

**Affiliations:** Department of Mechanical Engineering, National Cheng Kung University, Tainan 701, Taiwan

**Keywords:** triboelectric nanogenerators, surface roughness, charge transfer, laser ablation, pressure sensor

## Abstract

A triboelectric nanogenerator (TENG) holds significant potential as a self-powered pressure sensor due to its ability to convert mechanical energy into electrical energy. The output voltage of a TENG is directly correlated with the applied pressure, making it highly suitable for pressure sensing applications. Among the key factors influencing TENG performance, the microstructure on the surface plays a crucial role. However, the effect of surface microstructure on charge transfer behavior remains relatively underexplored. Here, a stripe-patterned rough TENG (SR-TENG) fabricated by laser ablation and molding is proposed. The stripe-patterned rough surface exhibits excellent deformation properties, allowing for more effective contact area between the tribolayers. Additionally, the localized surface-edge enhanced electric field at the stripe boundaries improves surface charge transfer, thereby enhancing overall output performance. The SR-TENG achieved an open-circuit voltage of 97 V, a short-circuit current of 59.6 μA, an instantaneous power of 3.55 mW, and a power density of 1.54 W/m^2^. As an energy harvester, the SR-TENG successfully powered 150 LEDs. A linear relationship between applied pressure and output voltage was established with a coefficient of determination R^2^ = 0.94, demonstrating a high sensitivity of 14.14 V/kPa. For practical application, a novel self-powered two-digit pressure switch was developed based on the SR-TENG. This system enables the control of two different LEDs using a single TENG device, triggered by applying a light or hard press.

## 1. Introduction

Pressure sensors play a vital role in daily life [1,2], industrial production [3,4], and medical care [5,6]. Based on their operating principles, pressure sensors can be classified into four main types: capacitive [7], resistive [8], piezoelectric [9], and triboelectric types [10,11]. With the rapid development of miniaturized sensors and advancements in computing capabilities, the demand for electronic sensors in everyday applications continues to rise [12]. However, capacitive and resistive pressure sensors typically require an external power supply. When deployed in large quantities, even though each sensor consumes only a small amount of energy, the total energy consumption can become significant. As a result, self-powered pressure sensors have attracted increasing attention [6]. Among self-powered types, piezoelectric sensors often involve complex fabrication processes or expensive materials, which may require additional steps such as phase transformation, electric polarization, or incorporation of nanofillers [13]. In contrast, pressure sensors based on triboelectric nanogenerators (TENG) offer several advantages, including low cost, simple fabrication, and a wide selection of material options. TENG operates based on the coupling of contact electrification and electrostatic induction [14]. Through frictional contact between two surfaces, charges are generated and transferred, continuously converting mechanical energy into electrical energy. Furthermore, the output voltage of a TENG is directly correlated with the applied pressure, making it particularly suitable for pressure sensing applications [14]. The output performance of a TENG depends primarily on two key factors: the selection of materials used as the triboelectric pair and the effective contact area of the triboelectric layer [15,16,17]. One promising research direction focuses on enhancing performance by introducing microstructures on the surface of the tribolayers to increase the effective contact area [18,19,20]. Examples of such microstructures are summarized in Table 1 [20,21,22,23,24,25,26,27]. For instance, Chung et al. fabricated PDMS micro-needle arrays using CO_2_ laser processing and polymer molding, achieving structures with heights around 1090 μm and densities of 388, 273, and 159 needles/cm^2^ [20], and Fan et al. used photolithography to create patterned silicon wafer molds, producing pyramid, square, and line-shaped arrays of 5 μm on PDMS surfaces [21]. However, these studies did not explicitly address the role of surface roughness in tribolayer performance. It has been demonstrated that increasing surface roughness can significantly enhance TENG output. For example, Song et al. utilized sandpaper with varying roughness as a master mold to create disordered microstructures on PDMS surfaces [26]. Mariello et al. introduced a steam-curing step to fabricate porous and rough PDMS substrates, improving output voltage [27]. Nevertheless, in these studies, the tribolayer surfaces were randomly rough, characterized by disordered and isotropic structures. The effect of regular and anisotropic rough surfaces on TENG output performance is still under development.

In this study, a stripe-patterned rough PDMS (denoted as SR-PDMS) was fabricated using CO_2_ laser ablation followed by a molding process [28]. The influence of the ordered and anisotropic surface roughness of SR-PDMS on the output performance of the TENG was systematically investigated. Based on the measured direction of charge transfer, it was found that the SR-PDMS exhibits an enhanced ability to accumulate surface charges. This behavior may be attributed to two combined factors: (1) The stripe-patterned rough surface exhibits excellent deformation properties, allowing for more effective contact area between the tribolayers, and (2) larger surface curvature variations at the edges of the stripe patterns, which promote stronger localized charge accumulation. As a result, the stripe-patterned rough surface of SR-PDMS plays a significant role in enhancing the output performance of the TENG. To the best of our knowledge, this is the first time that the effect of surface microstructure on charge transfer between two tribolayers made of the same material has been reported. In addition, SR-PDMS and aluminum foil are used as tribolayers for combining a SR-PDMS/Al TENG (denoted as SR-TENG) as a sensitive pressure sensor in low-pressure range. Owing to its high sensitivity to pressure, SR-TENG is employed as a self-powered two-digit switch for controlling two different LEDs using a single TENG device. Compared to conventional controllers that require external power and typically rely on one sensor to control a single actuator, the system presented in this paper uses one TENG to control two LEDs, offering greater flexibility and reduced energy consumption. With the growing importance of pressure sensors in the Internet of Things (IoT), such self-powered devices hold great potential for applications in telemedicine monitoring [29], fall detection [30], and intelligent musical instrument systems [31]. Benefiting from the self-powered functionality and high sensitivity of SR-TENG in the low-pressure range, the developed pressure sensor shows strong potential for future integration into electronic skin (e-skin) [32] and human–machine interaction systems [33].

## 2. Materials and Methods

**Figure 1:**
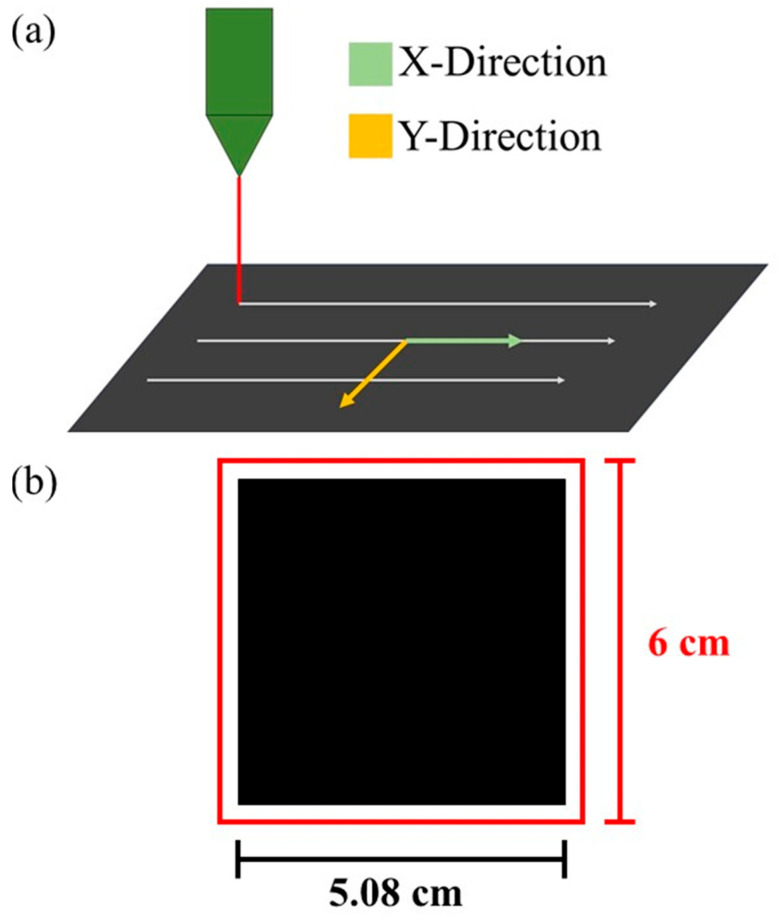
CO_2_ laser processing diagram: (**a**) Definition of the laser scanning directions: the X-direction is parallel to the laser processing path, while the Y-direction is perpendicular to it. (**b**) Schematic of the processed PMMA mold, where the red frame indicates the mold’s outer boundary, and the black square represents the laser-processed surface area.

The polymer material PMMA exhibits strong absorption at the CO_2_ laser wavelength (10.6 μm, VL-200, Universal Laser System Inc., Scottsdale, AZ, USA), making it suitable for direct laser processing. Figure 1 illustrates the CO_2_ laser processing setup. Figure 1a shows the laser scanning direction is defined as the X-direction, while the Y-direction is perpendicular to it. A mold with a laser-patterned rough surface (size: 5.08 × 5.08 cm^2^) was fabricated by ablating PMMA using a high-energy-density laser under the following parameters: 9 W laser power, 114 mm/s scanning speed, and 1000 points per inch (PPI). The laser beam mode is TEM_00_, and the size of the lens is 2.0”, which determines the depth of focus of the laser. The mold includes a 6 × 6 cm^2^ outer frame, as shown in Figure 1b. PDMS (Sylgard 184, Dow Corning) was prepared by mixing the elastomer base and curing agent at a 10:1 weight ratio. The mixture was poured into the PMMA mold, followed by vacuum degassing for 30 min to ensure complete filling of the microstructured cavities. Excess PDMS on the mold surface was then removed using a scraper. The mold was baked at 74 °C for two hours to cure the PDMS. After cooling to room temperature, the cured PDMS was demolded to obtain the stripe-patterned rough PDMS (SR-PDMS). The surface morphology of both PDMS and PMMA was characterized using optical microscopy (OM, Olympus BX51M, Hachiōji-shi, Tokyo, Japan) and a profilometer (Kosaka Surfcorder ET3000, Sotokanda Chiyoda-ku, Tokyo, Japan). For device assembly, the top and bottom substrates of the contact–separation TENG were fabricated from two 7 × 6 cm^2^PMMA plates, also processed by CO_2_ laser. Low-purity aluminum foil (6 × 6 cm^2^) was used as the electrode material and affixed to one side of each PMMA substrate. Four elastic spacers were prepared by cutting commercial sponge into 0.5 × 0.5 × 0.5 cm^3^ cubes and attaching them to the corners of the bottom substrate using double-sided tape. The SR-PDMS film was placed onto the bottom aluminum electrode, and the top PMMA substrate was positioned over the spacers to complete the TENG. Low-purity aluminum foil and PDMS were chosen as the tribopair materials due to their significant difference in triboelectric charge affinity. Additionally, both materials are low-cost and commercially available. Compared to other triboelectric materials such as PTFE, PDMS offers easier processing and molding. The TENG was driven by a pneumatic motor with a contact–separation distance of 25 mm and an operating frequency of 9 Hz. The open-circuit voltage (V_oc_) and short-circuit current (I_sc_) signals were recorded using an oscilloscope (HIOKI Memory HiCorder MR8870-20, Koizumi, Japan).

## 3. Results and Discussion

### 3.1. Laser Processing Rough Surface Morphology

**Figure 2:**
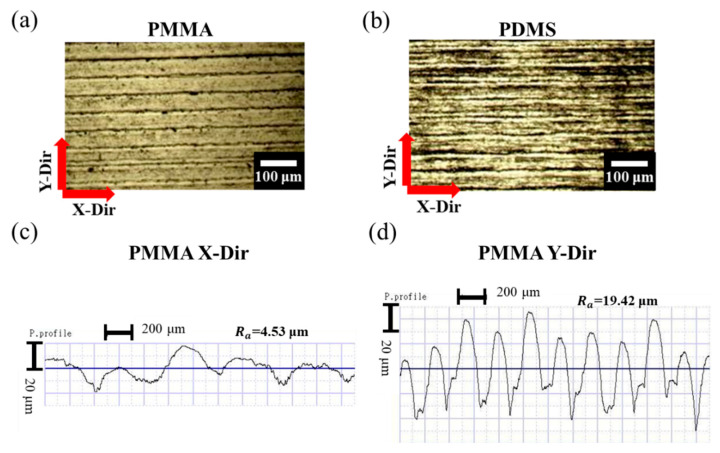
Surface morphology measurement of PMMA mold and SR-PDMS: Top view of (**a**) PMMA mold and (**b**) SR-PDMS under an optical microscope. PMMA surface morphology in (**c**) X and (**d**) Y directions under profilometer, respectively.

To investigate the actual surface morphology of the tribolayer fabricated through laser ablation and molding, the optical microscope images and profilometer measurements are presented in Figure 2. Figure 2a,b show the top-view optical microscope images of (a) the PMMA mold fabricated by CO_2_ laser ablation and (b) the stripe-patterned rough PDMS (SR-PDMS) surface replicated from the PMMA mold via molding. The laser ablation process creates well-defined and regularly spaced stripe-patterned grooves on the PMMA mold. After molding, the SR-PDMS surface exhibits an inverse replication of the PMMA mold’s microstructure. Figure 2c,d display profilometer measurements of the PMMA mold surface taken along the X and Y directions, respectively. The average surface roughness in the X-direction (parallel to the grooves), denoted as R_ax_, is measured to be 4.53 μm, while the average roughness in the Y-direction (perpendicular to the grooves), denoted as R_ay_, is 19.42 μm, respectively. The profile along the X-direction indicates that the groove bottoms formed by laser ablation are not perfectly smooth, likely due to thermal effects induced by the laser. In contrast, the Y-direction profile, which traverses multiple grooves, clearly shows the depth of the grooves, with an average peak-to-valley height difference of approximately 78 μm. In summary, the SR-PDMS tribolayer can be effectively fabricated using a straightforward combination of laser ablation and molding. The resulting surface consists of a stripe grooves array with pronounced anisotropic roughness, which may contribute significantly to its triboelectric performance. In addition, according to the previously published literature [20], an array of micro-needled PDMS (denoted as MN-PDMS) was also prepared as a control group. The OM image of the micro-needle array is shown in Figure S1, and the height of the micro-needles is approximately 1241.7 μm.

### 3.2. The Output Performance of SR-TENG

**Figure 3:**
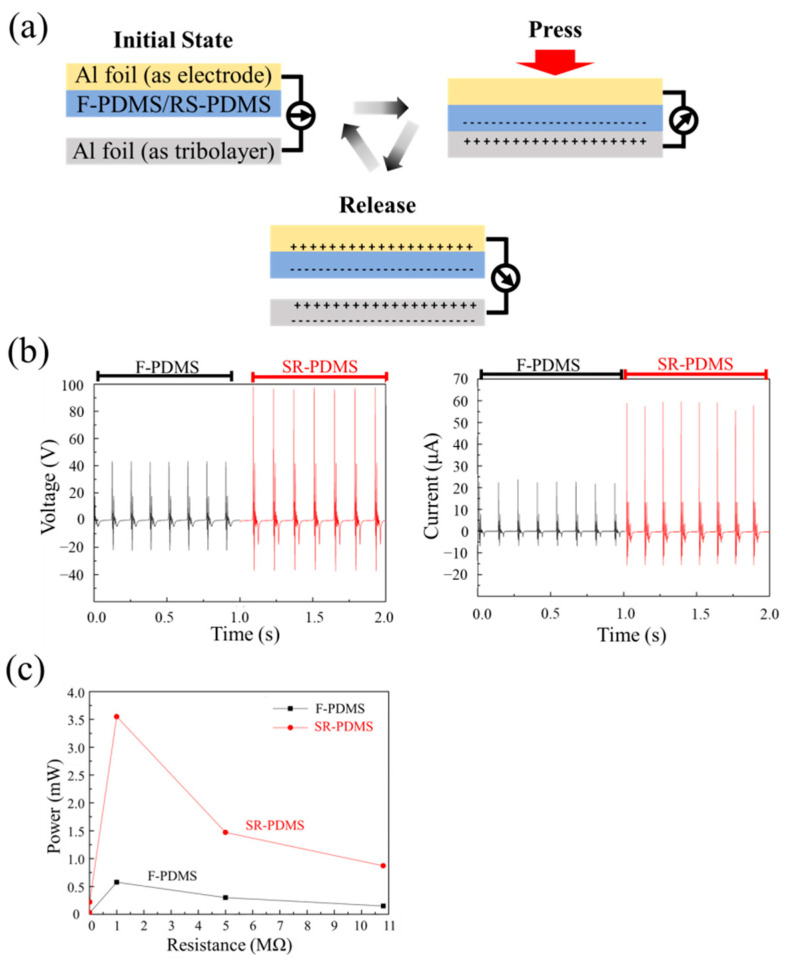
(**a**) Schematic diagram of the cyclic operation of the vertical contact–separation TENG and its charge distribution. (**b**) Measurement of open-circuit voltage and short-circuit current of F-PDMS and SR-PDMS: voltage (**left** panel) and current (**right** panel) were measured during real-time using a pneumatic motor. (**c**) Maximum instantaneous power trend of F-PDMS and SR-PDMS under different loads of 10 Ω, 1 kΩ, 10 kΩ, 1 MΩ, 5 MΩ, and 10.8 MΩ, respectively.

To evaluate the performance of the SR-TENG, a flat PDMS surface is used as a reference for comparison. Figure 3a presents a schematic diagram illustrating the cyclic operation and charge distribution of a vertical contact–separation TENG. In this configuration, either flat PDMS (F-PDMS) or SR-PDMS is paired with aluminum as the triboelectric layers. An additional aluminum foil layer, connected to the PDMS side, serves as the electrode for measuring the output voltage and current of the TENG device. The role of the aluminum foil, whether as a triboelectric layer or merely as an electrode, depends on its interaction with the PDMS surface. If the aluminum undergoes periodic contact and separation with the PDMS layer, it functions as a triboelectric layer. In contrast, if it remains stationary and does not engage in contact–separation motion, it acts solely as an electrode. Initially, the tribopair is in a separate state. When external force is applied, the aluminum film comes into contact with the PDMS surface, leading to charge transfer from the aluminum to the PDMS. As a result, equal and opposite charges are generated on the two tribolayers, with the aluminum foil carrying positive charges and the PDMS carrying negative charges. Moreover, as charge transfers between the two triboelectric layers, a potential difference is generated between them, resulting in the formation of voltage and current in the external circuit. When the two tribolayer layers are in full contact, the voltage reaches its peak value. Furthermore, as current flows through the external circuit, the voltage generated by the triboelectric effect in the TENG gradually decreases. When the two triboelectric layers begin to separate, the aluminum foil becomes positively charged, while the PDMS layer carries negative charges. These charges induce opposite charges on the conductive layers, negative on the opposite side of the aluminum foil and positive on the aluminum foil (as electrode) connected to the PDMS. This charge distribution creates a new potential difference between the two electrodes, once again generating voltage and current in the external circuit. It is noticed that the direction of the voltage generated during separation is opposite to that generated during contact. This cyclic contact–separation process continuously converts mechanical motion into alternating electrical output.

Figure 3b shows the open-circuit voltage (V_oc_) and short-circuit current (I_sc_) of F-PDMS and SR-PDMS actuated by a pneumatic motor at an input pressure of 2 kg/cm^2^. The average open-circuit voltages generated by F-PDMS and SR-PDMS are 44.1 V and 97 V, respectively. Similarly, their short-circuit currents are measured to be 23.7 μA and 59.6 μA, respectively. Compared to F-PDMS, SR-PDMS exhibits approximately 2.2 times higher open-circuit voltage and 2.5 times higher short-circuit current, indicating a significant enhancement in electrical output performance due to the stripe-patterned rough surface structure. Figure S2 shows the voltage signals from a single contact–separation cycle between (a) F-PDMS, (b) SR-PDMS, and (c) MN-PDMS with aluminum. The voltage vibrations observed in Figure S2 can be attributed to the oscillation of the spring inside the TENG device during the separation phase. Since TENG devices are highly sensitive systems, even slight vibrations can generate voltage signals. When the pneumatic motor disengages from the TENG setup, the residual spring oscillations may lead to the subsequent voltage signals.

In Figure 3b and Figure S2, the voltage asymmetry originates from the material selection and inherent properties of the system. Specifically, the first observed peak and the subsequent reverse peak arise from different underlying mechanisms. The initial peak is attributed to charge transfer generated by the friction between the two tribolayers, whereas the second peak results from the induced potential formed as the tribolayers separate. Several literatures have investigated the mechanisms underlying the voltage asymmetry in contact–separation mode TENGs [34,35,36]. For instance, Chen et al. attributed the voltage asymmetry to the asymmetry in contact–separation velocity [34]; Wang et al. discussed the asymmetric change in capacitance during the contact–separation process [35]; and Dharmasena explored the influence of the sticking effect between two tribolayers on voltage asymmetry [36]. However, most of these studies suggest that the voltage generated during the separation phase is greater than that during the contact phase, which is inconsistent with the phenomenon observed in this work. However, similar behavior, where a higher voltage is generated during contact and a lower voltage during separation, has been widely observed in several other reports [37,38,39]. Therefore, a comprehensive explanation for this phenomenon is still under development.

Figure 3c presents the maximum instantaneous power outputs of both F-PDMS and SR-PDMS under various load resistances of 10 Ω, 1 kΩ, 10 kΩ, 1 MΩ, 5 MΩ, and 10.8 MΩ. Both configurations achieve peak power output at a load of 1 MΩ, yielding 0.58 mW for F-PDMS and 3.55 mW for SR-PDMS. When normalized by the area of the tribolayers, the corresponding power densities of F-PDMS and SR-PDMS are calculated to be 0.25 W/m^2^ and 1.54 W/m^2^, respectively. Overall, the maximum instantaneous power of SR-PDMS is approximately 6.16 times greater than that of F-PDMS. From the friction test, it can be observed that fabricating a stripe-patterned rough structure on the surface of PDMS, as compared to a completely flat structure, helps to improve the output performance. Traditionally, the performance enhancement resulting from such microstructures has been attributed to the increase in effective contact area provided by the microstructures. However, as shown in Table 1, under the same choice of triboelectric materials (both using Al/PDMS), the stripe-patterned rough structure proposed in this study exhibits a comparable voltage output to that of the conventional microneedle array structure, even though the effective contact area of the microneedle array is significantly larger than that of the stripe-patterned rough structure. So, in addition to the effective contact area, there is clearly another mechanism that allows the stripe-patterned rough structure to significantly enhance the output voltage.

### 3.3. The Influence of the Surface Roughness of the Tribolayer on Triboelectricity

**Figure 4:**
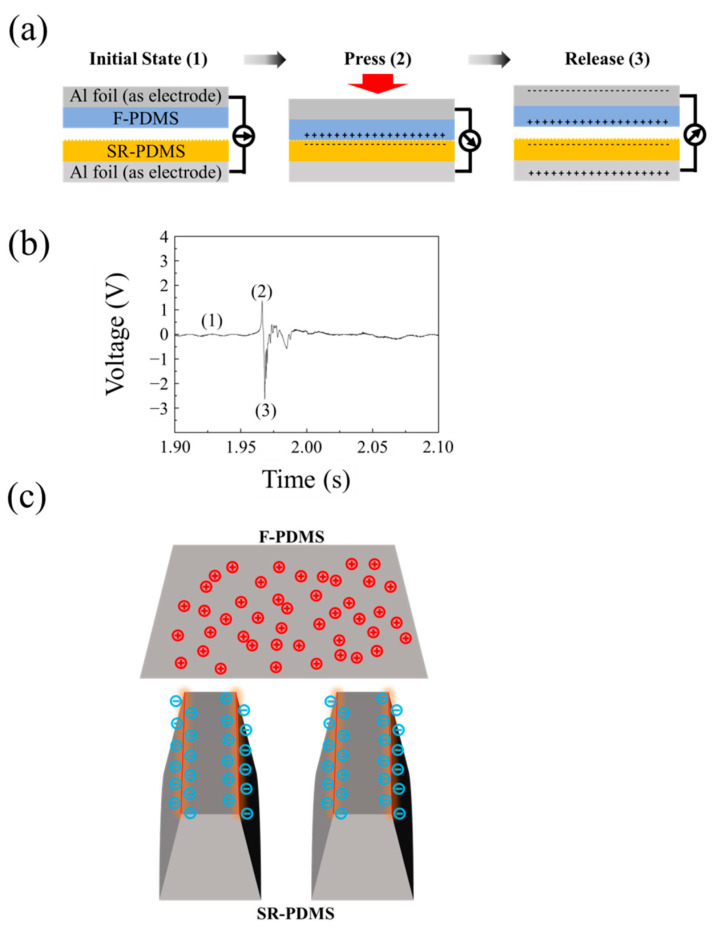
(**a**) Schematic diagram of the vertical contact–separation process between F-PDMS and SR-PDMS. (**b**) Voltage signal generated from a single contact–separation cycle. (**c**) Schematic illustration of the charge transfer behavior between F-PDMS and SR-PDMS.

To further investigate the influence of stripe-patterned roughness on the output performance of tribolayer surfaces made from the same material, both the stripe-patterned rough tribolayer and the flat tribolayer were fabricated using identical PDMS material. This ensures that the only variable between the two is the surface structure. Vertical contact–separation interactions were performed between the SR-PDMS and the F-PDMS, and the charge transfer between the SR-PDMS and F-PDMS surfaces was measured during the contact–separation process. Figure 4 shows (a) the schematic diagram of the charge transfer behavior between the SR-PDMS and F-PDMS and (b) the measured voltage signal between the F-PDMS and SR-PDMS during a single contact–separation cycle. At the initial stage (Step 1), the two tribolayers are fully separated, and no voltage or current is generated. When an external force is applied (Step 2), the F-PDMS and SR-PDMS surfaces come into contact, leading to triboelectric charge transfer. Positive charges accumulate on the F-PDMS side, while negative charges form on the SR-PDMS side, resulting in a positive voltage signal. Upon release of the force (Step 3), the tribolayers separate, and electrostatic induction drives electrons through the external circuit, generating a negative voltage signal. Since both tribolayers are made from the same base material (PDMS), the observed charge transfer is attributed to differences in the surface microstructure between the two layers. Based on the measured direction of charge transfer, it was found that the surface structure of SR-PDMS exhibits an enhanced ability to accumulate surface charges. Figure S3 shows an additional experimental set that included micro-needle structured PDMS (MN-PDMS) as one of the tribolayers. Experiments by pairing three sets of surface morphologies—(a) MN-F, (b) SR-F, and (c) SR-MN—are conducted. The results shown in Figure S3a,b indicate that when a structured surface is in frictional contact against a flat surface, the charge flow direction remains the same. Specifically, a positive voltage is generated upon contact, followed by a negative voltage upon separation. The difference between Figure S3a,b lies in the magnitude of the voltage, which quantitatively reflects the charge transfer capability. The data show that while MN-F and SR-F generate similar positive voltages, SR-F exhibits a significantly larger negative voltage compared to MN-F. This suggests that SR-F possesses superior charge transfer ability. In the SR-MN pairing shown in Figure S3c, since the SR surface demonstrates better charge transfer capability, the voltage behavior follows a similar pattern to that of MN-F and SR-F, generating a positive voltage first, followed by a negative voltage. However, because both SR and MN surfaces feature microstructures, the voltage generated during their frictional interaction is reduced to about one-half to two-thirds of the values observed in MN-F and SR-F. This observation suggests that when two tribolayers with microstructured surfaces are in frictional contact against each other, the resulting voltage is actually lower than when only one structured tribolayer interacts with a flat tribolayer. These findings indicate that charge transfer is indeed influenced by surface morphology. Specifically, structured surfaces exhibit enhanced electron acquisition ability. However, when both tribolayers have microstructures and simultaneously compete for electrons, the friction-induced potential becomes lower than in cases where only one structured surface is involved. Using the same material while altering only the surface structure, the resulting voltage demonstrates that surface morphology can influence electron affinity. It is worth noting that, as shown in Figure S2b,c, although the output voltage of the Al/MN-PDMS combination was higher than that of the Al/SR-PDMS. However, for the TENG involving aluminum and PDMS, the output voltage is influenced not only by electron affinity but also by the effective contact area. Therefore, although the Al/MN-PDMS (119.2 V and −52.4 V) exhibited higher output voltage compared to the Al/SR-PDMS (97 V and −41.6 V), this does not necessarily imply that MN-PDMS has a higher position in the triboelectric series. In contrast, the result in Figure S3c indicates that SR-PDMS possesses a better electron affinity compared to MN-PDMS. The high electron affinity of SR-PDMS is demonstrated in Figure 4c.

Figure 4c shows a schematic diagram of how the SR-patterned surface influences the charge transfer behavior. This effect is likely due to two combined factors: (1) The stripe-patterned rough surface exhibits excellent deformation properties, allowing for more effective contact area between the tribolayers, and (2) larger surface curvature variations at the edges of the stripe patterns, which promote stronger localized charge accumulation. As a result, the voltage signal indicates that charge transfer occurs from the F-PDMS side to the SR-PDMS side during the contact–separation cycle. To the best of our knowledge, this is the first reported observation of charge transfer behavior between two triboelectric layers composed of the same material but differing in surface microstructure.

**Figure 5:**
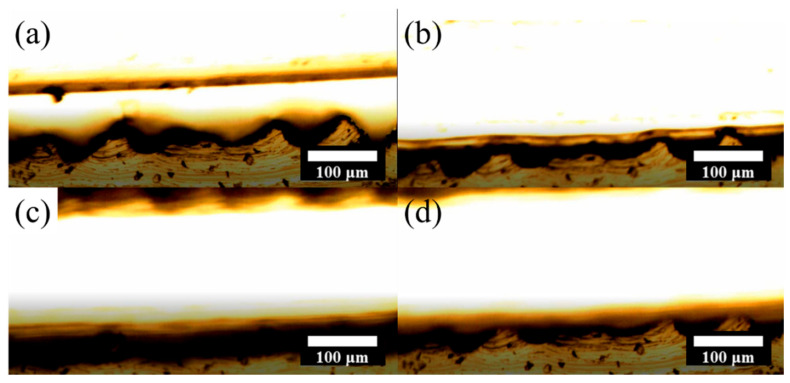
Optical microscope cross-sectional images of F-PDMS and SR-PDMS during a contact–separation cycle: (**a**) Initial state where the tribopair is fully separated with no contact. (**b**) Upon contact, frictional interaction occurs between the surfaces. (**c**) At full contact, SR-PDMS undergoes noticeable deformation, consuming more frictional energy and facilitating charge transfer from F-PDMS to SR-PDMS. (**d**) The tribopair returns to the initial separated state as the external force is released.

Furthermore, the deformation of the surface structure of both tribolayers in a single contact–separation cycle is observed by an optical microscope. Figure 5 shows the deformation process of the SR-PDMS surface structure under varying pressure conditions. Figure 5a shows the initial separation state. As pressure is gradually applied, Figure 5b demonstrates that the F-PDMS begins to make contact with the SR-PDMS surface. Under maximum external force, the tribopair reaches full contact, and Figure 5c reveals that the SR-PDMS microstructure deforms and conforms closely to the F-PDMS surface. When the external force is released, Figure 5d shows the elastic recovery of the SR-PDMS structure as the two layers separate, returning to its original form due to its inherent material properties. These observations reveal that during frictional contact between F-PDMS and SR-PDMS, the fine microstructures of SR-PDMS undergo vertical deformation. A portion of the mechanical energy from friction is thus consumed in deforming the surface structure. As a result, F-PDMS may retain higher surface energy during contact, leading to preferential electron transfer from the F-PDMS to the SR-PDMS. Additionally, the stripe-patterned surface of SR-PDMS exhibits higher curvature at the edges, which facilitates localized charge accumulation and stronger electric fields. This effect further enhances the surface charge density and contributes to improved triboelectric performance. Due to the larger surface curvature variations at the edges of the stripe structures lead to better surface charge density, and the SR-PDMS could be a good potential candidate as a pressure sensor.

### 3.4. The SR-PDMS for Energy Harvesting

**Figure 6:**
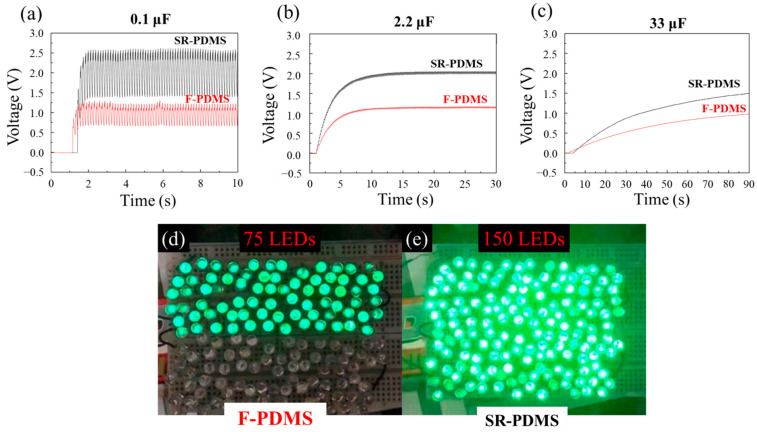
The comparison of F-PDMS and SR-PDMS in energy storage in capacitors and lighting up LEDs: The charging voltage curve on a (**a**) 0.1 μF, (**b**) 2.2 μF, (**c**) 33 μF capacitor and LED lighting application of (**d**) F-PDMS driving 75 LEDs and (**e**) SR-PDMS driving 150 LEDs.

TENG can function as an energy harvester and serve as a power source in electronic circuits [40,41]. To assess its energy output capabilities, a pneumatic motor was used as the actuation platform, operating at a fixed input air pressure of 2 kg/cm^2^. The charging performance of F-PDMS and SR-PDMS was evaluated using three different capacitors and LED arrays, and the results are presented in Figure 6. Figure 6a–c display the charging curves for capacitors with capacitances of 0.1 μF, 2.2 μF, and 33 μF, respectively. Figure 6a shows that F-PDMS charges a 0.1 μF capacitor to a saturation voltage of 1.4 V, while SR-PDMS reaches 2.6 V within 1 s. Figure 6b reveals that a 2.2 μF capacitor charges to 1.0 V with F-PDMS and 2.1 V with SR-PDMS in 8 s. Figure 6c demonstrates that a 33 μF capacitor is charged to 0.99 V by F-PDMS and 1.55 V by SR-PDMS within 90 s. For the same capacitance, a higher saturation voltage indicates greater charge transfer during the contact–separation cycle. These results confirm that the laser-processed, stripe-patterned rough surface of SR-PDMS significantly enhances the surface charge transfer on the PDMS layer of the TENG, leading to improved energy storage performance in capacitors. The difference in performance is further reflected in LED-driving capability. Figure 6d demonstrates that F-PDMS lights up 75 green LEDs, while Figure 6e reveals that SR-PDMS powers up to 150 green LEDs, twice as many as those lightened by F-PDMS. This enhancement is primarily attributed to the increased surface charge transfer resulting from the stripe-patterned roughness of SR-PDMS, which provides a higher instantaneous power output under the same load. The triboelectric-powered LED arrays exhibit strong potential for self-powered applications in lighting systems [42,43], visual signal warning systems [44], and display devices [45].

### 3.5. The SR-PDMS as a Pressure Sensor and Used as a Two-Digit Switch

**Figure 7:**
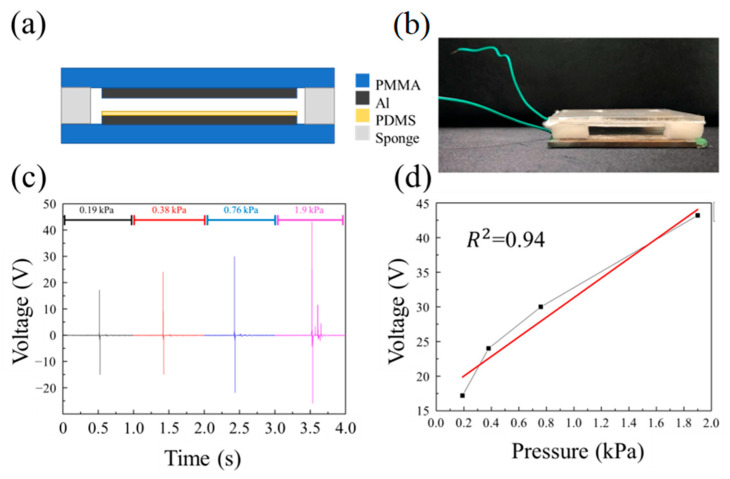
(**a**) Schematic diagram of a SR-TENG. (**b**) Side view of an actual photograph of a SR-TENG. (**c**) The voltage signals of the TENG that were generated by various pressure of 0.19, 0.38, 0.76, and 1.9 kPa, respectively. (**d**) Linear fitting of the voltage and the external pressure.

Taking advantage of the correlation between the output voltage of the TENG and the applied load, SR-PDMS is applied in TENG-based self-powered pressure sensors. In addition, the self-powered nature of TENGs, which do not require an external power source like resistive or capacitive pressure sensors, makes them highly suitable for such applications. Figure 7 shows the actual photograph of the SR-TENG and its output performance under different loads. Figure 7a shows the schematic of the device structure, and Figure 7b shows an actual photograph of the SR-TENG device. To evaluate its pressure sensitivity, various weights of 50 g, 100 g, 200 g, and 500 g were dropped from a fixed height of 5 cm onto the SR-TENG, corresponding to pressures of 0.19 kPa, 0.38 kPa, 0.76 kPa, and 1.9 kPa, respectively. Figure 7c shows the resulting voltage outputs of 17.2 V, 24 V, 30 V, and 43.2 V generated by the different pressures of 0.19 kPa, 0.38 kPa, 0.76 kPa, and 1.9 kPa, respectively. Figure 7d plots the linear fitting curve of the pressure-voltage response, yielding a calculated sensitivity of 14.1 V/kPa with a coefficient of determination (R^2^) of 0.94. The SR-TENG offers several advantages, including direct force transmission, a short contact–separation cycle, and a simple mechanical design. It exhibits high mechanical-to-electrical energy conversion efficiency and can generate significant voltage signals under low pressure.

Table 2 lists reported pressure sensors based on TENG and their morphology, tribolayer materials, measuring range, and sensitivity [18,46,47,48]. Compared to another TENG composed of aluminum foil and PDMS with a microneedle structure [18], the SR-TENG exhibits much higher sensitivity, about 4.5 times. This comparison highlights the excellent charge transfer characteristics of the stripe-patterned rough microstructure, resulting in outstanding pressure sensing sensitivity. Furthermore, in comparison with other structures fabricated through more complex processes, such as micro-pyramids [37] or nanorods [47], the stripe-patterned rough microstructure still demonstrates superior sensitivity. In addition, compared to complex and expensive two-dimensional materials like crumpled MXene [48], the stripe-patterned rough structure presented in this study offers not only higher sensitivity but also a broader detection range. Through these lateral comparisons, it is evident that the stripe-patterned rough structure proposed in this work outperforms most triboelectric-based pressure sensors. These results indicate that SR-TENG holds strong potential for use in applications requiring high-sensitivity and precision vibration sensing, such as wearable health monitoring systems [49] or human motion detection [50].

**Figure 8:**
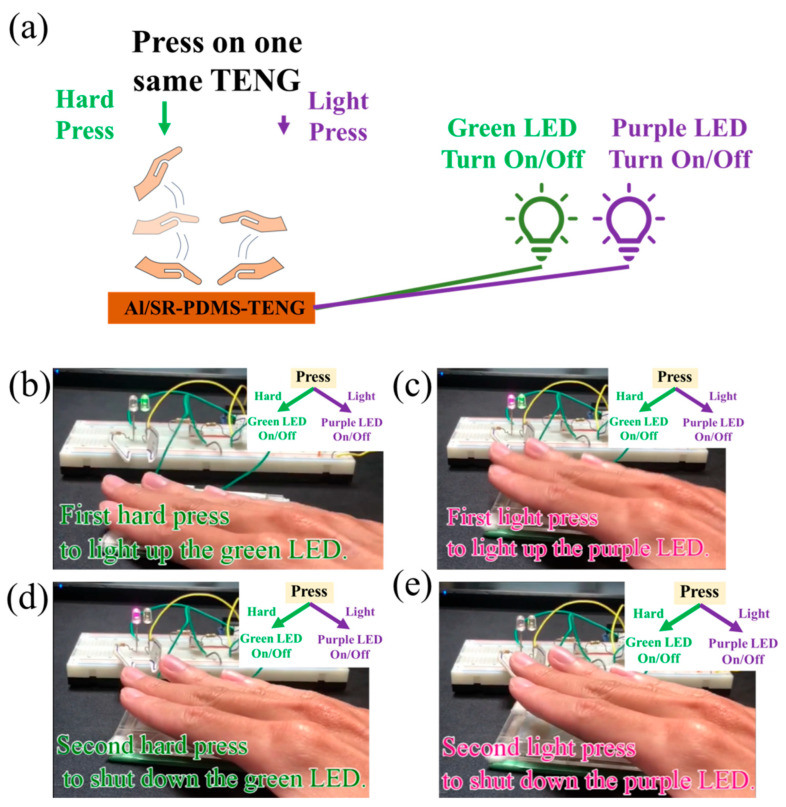
(**a**) The schematic of the use of one single SR-TENG to achieve two-digit control for turning on and off two different LEDs. (**b**,**c**) When the first (**b**) hard and (**c**) light press is applied, the (**b**) green LED and (**c**) purple LED are activated, respectively. And then, (**d**,**e**) when the second (**d**) hard and (**e**) light press is applied, the (**d**) green LED and (**e**) purple LED are deactivated.

By combining the SR-TENG with the programmable Arduino UNO, the SR-TENG is utilized as a self-powered two-digit switch capable of controlling two different LEDs with a single TENG device. Figure 8 shows the schematic diagram and actual photographs of the self-powered two-digit switch along with its working cycle. Figure 8a shows the schematic diagram that the SR-TENG converts applied pressure into two distinct digital signals corresponding to a hard press and a light press. These two pressure levels are used to control the on/off states of the green and purple LEDs, respectively. Specifically, the hard press exclusively triggers the green LED, while the light press controls only the purple LED. When the first (b) hard and (c) light press are applied, the (b) green LED and (c) purple LED are activated, respectively. And then, when the second (d) hard and (e) light press are applied, the (d) green LED and (e) purple LED are deactivated. Compared to conventional switches that typically control only one actuator [51], this triboelectric pressure device functions as a two-digit switch, enabling the control of two outputs using a single TENG through pressure differentiation. It features a simple mechanism, fast response, ease of operation, and minimal system complexity. Furthermore, it operates reliably across various environments without the constraints of conventional control systems. This approach provides a simplified and integrated design concept for multi-switch mechanisms in everyday applications and shows strong potential for future integration into flexible electronics and electronic skin (e-skin) systems [52].

## 4. Conclusions

A laser-processed stripe-patterned rough surface structure on PDMS is proposed, offering a low-cost and simple fabrication method that effectively enhances the output performance of TENG devices. The performance improvement can be attributed to the stripe-patterned rough surface exhibiting excellent deformation properties and the surface curvature variations of the stripe edge, which increase surface roughness and promote more efficient charge transfer. The open-circuit voltage (V_oc_) and short-circuit current (I_sc_) of SR-PDMS reach 97 V and 59.6 μA, respectively, approximately 2.2 and 2.5 times higher than those of F-PDMS (44.1 V and 23.7 μA). In terms of practical applications, the SR-TENG functions as a high-sensitivity pressure sensor with a sensitivity of 14.1 V/kPa within a low-pressure range of 0.19 to 1.9 kPa. In addition, SR-TENG can also serve as a power source, capable of charging capacitors and powering up to 150 LEDs. Furthermore, by integrating SR-TENG with an Arduino UNO, a self-powered two-digit pressure-sensing switch is demonstrated, capable of controlling two LEDs with one single TENG device. This research highlights the potential of SR-TENG for future integration into TENG-based electronic skin (e-skin) systems, offering designers more versatile and practical solutions for wearable electronics and human–machine interfaces.

## Figures and Tables

**Table 1:** Morphologies, parameters, fabric methods, and the TENG output performance of tribolayers.

Morphology	Surface Roughness R_a_ (μm)	Triboelectric Material	Fabrication Method	Open-Circuit Voltage (V)	Short-Circuit Current (μA)	Instantaneous Power Density (W/m^2^)	Ref.
Needle	Not mentioned	Al-PDMS	CO_2_ laser ablation	102.8	43.1	-	[20]
Pyramid		PET-PDMS	Photolithography	18	0.7	-	[21]
Cube				<18	<0.7	-	
Line				<18	<0.7	-	
Nanodots		BCP-PTFE	Self-assembled BCP	95	2.16	0.09	[22]
Nanogrates				110	2.76	0.104	
Crumpled		Au-PDMS	Spin coating	75.3	7.4	2	[23]
Tiny burr arrays		CB@PDMS-PDMS	CO_2_ laser ablation	70	**-**	**-**	[24]
Semi-ordered micro-sized structures		FEP-Nylon	Hot embossing	30	7.5	0.0157	[25]
Rough	10.9 (Random rough)	ACF-PDMS	Molding of sandpaper	80.4	11.8	1.9	[26]
Porous	30–40 (Random rough)	Au/Ti-PDMS	Steam-curing	1.6	0.15	0.00224	[27]
Stripe Rough	12 (Regular rough)	Al-PDMS	CO_2_ laser ablation	97	59.6	1.54	This article

**Table 2:** Comparison of the sensitivity and measurement interval of the TENG-based pressure sensor in each study.

Morphology	Roughness (Ra)	Triboelectric Material	Fabrication Method	Measuring Range (kPa)	Sensitivity (V/kPa)	Ref
Needle	No mentioned	Al-PDMS	CO_2_ laser ablation and molding	0.2~2.4	3.11	[18]
Rough	13.2 nm	polymer-PBS/SR	Surface self-modified sustainable polymer	2.1~62	0.135	[46]
Micro-pyramid	Not mentioned	Human skin-PDMS	Etching and molding of silicon wafer	0.4~7.3	0.29	[37]
Nanorod	Not mentioned	Cu-FEP	top-down dry etching on FEP membrane	3.1–9.4	3.2	[47]
Crumpled MXene	Not mentioned	MXene-PET	crumpled structured MXene	0.3–1	2.35	[48]
Stripe-Patterned Rough	12 μm (Regular rough)	Al-PDMS	CO_2_ laser ablation and molding	0.19~1.9	14.14	This article

## Data Availability

Data are presented in the coauthors’ research results, and the schematic drawing is available upon request.

## Supplementary Materials

The following supporting information can be downloaded at: https://www.mdpi.com/article/10.3390/nano15100760/s1, Figure S1: The OM of the micro-needle PDMS as a tribolayer.; Figure S2: Voltage signals generated by a single contact-separation movement of the tribolayers are (a) Al/F-PDMS (41.4 V/-19.3 V), (b) Al/SR-PDMS (97 V/-41.6 V), and (c) Al/MN-PDMS (119.2 V/-52.4 V), respec-tively.; Figure S3: Voltage signals generated by a single contact-separation movement of the tribolayers are (a) MN-F (1.5V/-1 V), (b) SR-F (1.45V/-2.6 V), and (c) SR-MN(0.75V/-1.7 V), respectively.
