# Supplementary material for: Stripe-Patterned Al/PDMS Triboelectric Nanogenerator for a High-Sensitive Pressure Sensor and a Novel Two-Digit Switch with Surface-Edge Enhanced Charge Transfer Behavior
Source: Nanomaterials (Basel). 2025 May 19;15(10):760. doi: 10.3390/nano15100760 (PMC12113814; doi:10.3390/nano15100760)
Supplement: Supplementary file 1 [file nanomaterials-15-00760-s001.zip › nanomaterials-3599197-supplementary.pdf]

nanomaterials

MDPI

Supplementary Materials

Stripe-patterned Al/PDMS triboelectric nanogenerator for high-sensitive pressure sensor and a novel two-digit switch with surface-edge enhanced charge transfer behavior

Chung-Yu Yu, Chia-Chun Hsu, Chin-An Ku and Chen-Kuei Chung*

Department of Mechanical Engineering, National Cheng Kung University, Tainan 701, Taiwan;
* Correspondence: ckchung@mail.ncku.edu.tw

According to previously published literature [1], a micro-needle array was also prepared as a control group. The OM image of the micro-needle array is shown in Figure S1, and the height of the micro-needles is approximately 1241.7 μm.

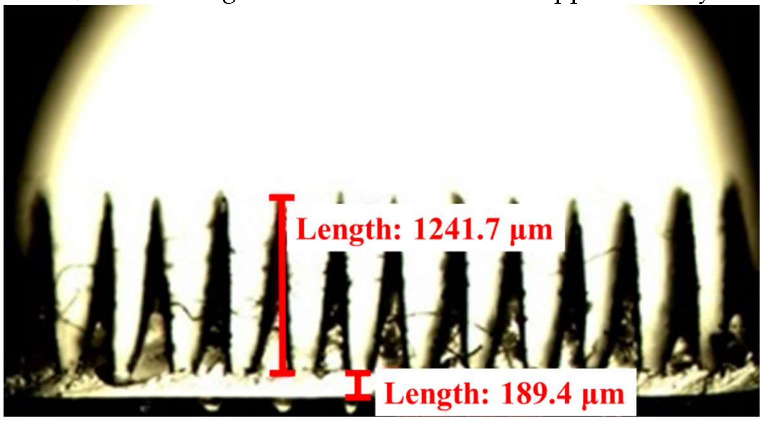

Figure S1 The OM of the micro-needle PDMS as a tribolayer.

Figure S2 shows the voltage signals from a single contact-separation cycle between (a) F-PDMS, (b) SR-PDMS, and (c) MN-PDMS with aluminum. The voltage vibrations observed in Figure S2 can be attributed to the oscillation of the spring inside the TENG device during the separation phase. Since TENG devices are highly sensitive systems, even slight vibrations can generate voltage signals. When the pneumatic motor disengages from the TENG setup, the residual spring oscillations may lead to the subsequent voltage signals. The voltage asymmetry originates from the material selection and inherent properties of the system. Specifically, the first observed peak and the subsequent reverse peak arise from different underlying mechanisms. The initial peak is attributed to charge transfer generated by the friction between the two tribolayers, whereas the second peak results from the induced potential formed as the tribolayers separate..

It is worth noting that, although the output voltage of the Al/MN-PDMS combination was higher than that of the Al/SR-PDMS. However, for the TENG involving aluminum and PDMS, the output voltage is influenced not only by electron affinity but also by the effective contact area. Therefore, although the Al/MN-PDMS (119.2 V and -52.4 V) exhibited higher output voltage compared to the Al/SR-PDMS (97 V and -41.6 V), this does not necessarily imply that MN-PDMS has a higher position in the triboelectric series.

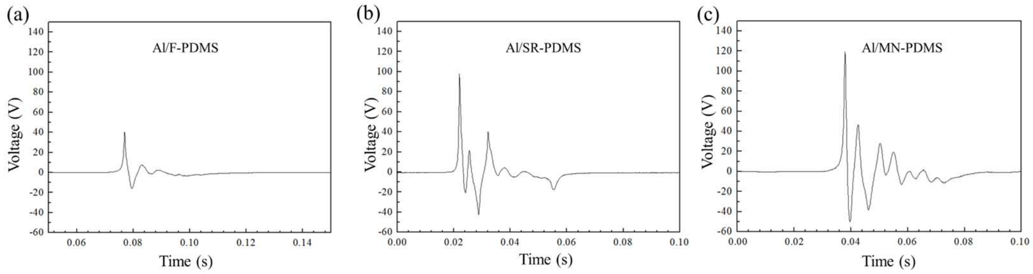

Figure S2 Voltage signals generated by a single contact-separation movement of the tribolayers are (a) Al/F-PDMS (41.4 V/-19.3 V), (b) Al/SR-PDMS (97 V/-41.6 V), and (c) Al/MN-PDMS (119.2 V/-52.4 V), respectively.

Figure S3 shows an additional experimental set that included micro-needle structured PDMS (MN-PDMS) as one of the tribolayers. Experiments by pairing three sets of surface morphologies (a) MN-F, (b) SR-F, and (c)SR-MN are conducted. The results shown in Figure S3 (a) and (b) indicate that when a structured surface is frictional contact against a flat surface, the charge flow direction remains the same. Specifically, a positive voltage is generated upon contact, followed by a negative voltage upon separation. The difference between Figure S3 (a) and (b) lies in the magnitude of the voltage, which quantitatively reflects the charge transfer capability. The data show that while MN-F and SR-F generate similar positive voltages, SR-F exhibits a significantly larger negative voltage compared to MN-F. This suggests that SR-F possesses superior charge transfer ability. In the SR-MN pairing shown in Figure S3 (c), since the SR surface demonstrates better charge transfer capability, the voltage behavior follows a similar pattern to that of MN-F and SR-F, generating a positive voltage first, followed by a negative voltage. However, because both SR and MN surfaces feature microstructures, the voltage generated during their frictional interaction is reduced to about one-half to two-thirds of the values observed in MN-F and SR-F. This observation suggests that when two tribolayers with microstructured surfaces are frictional contact against each other, the resulting voltage is actually lower than when only one structured tribolayer interacts with a flat tribolayer. These findings indicate that charge transfer is indeed influenced by surface morphology. Specifically, structured surfaces exhibit enhanced electron acquisition ability. However, when both tribolayers have microstructures and simultaneously compete for electrons, the friction-induced potential becomes lower than in cases where only one structured surface is involved. Using the same material while altering only the surface structure, the resulting voltage demonstrates that surface morphology can influence electron affinity.

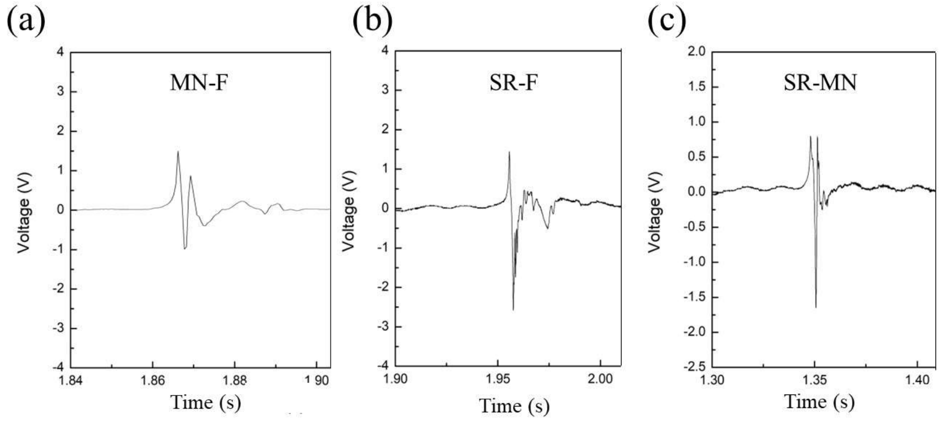

Figure S3 Voltage signals generated by a single contact-separation movement of the tribolayers are (a) MN-F (1.5V/-1 V), (b) SR-F (1.45V/-2.6 V), and (c) SR-MN(0.75V/-1.7 V), respectively.

Reference:

1. V. L. Trinh, & C. K. Chung, "A Facile Method and Novel Mechanism Using Microneedle-Structured PDMS for Triboelectric Generator Applications," Small, 13(2017),1700373.
